# Predictors of COVID-19 Related Stress Among Custodial Grandmothers

**DOI:** 10.1093/geroni/igab046.317

**Published:** 2021-12-17

**Authors:** Alexandra Jeanblanc, Megan Dolbin-MacNab, Carol Musil, Gregory Smith

**Affiliations:** 1 Case Western Reserve University, Cleveland, Ohio, United States; 2 Virginia Polytechnic University, Blackburg, Virginia, United States; 3 CWRU School of Nursing, Cleveland, Ohio, United States; 4 Kent State University, Kent, Ohio, United States

## Abstract

This paper examined predictors of COVID-19 stressors among 316 custodial grandmothers raising school-aged grandchildren using regression. Grandmothers, who were participants in two nationwide behavioral RCTs, completed an online questionnaire in Spring 2020. Predictors included grandmother demographics, depressive symptoms, perceived caregiving stress and reward, stress management strategies, and grandchild factors. Outcomes included grandmothers’ stress related to using bad coping habits (r2=.24), grandchildren’s remote learning(r2=.39), household conflict (r2=.29), COVID-19 fear and uncertainty (r2=.28), and finances(r2=.24). Regression results indicated that grandmothers’ pre-existing depressive symptoms predicted all outcomes except remote learning stress. Higher caregiving stress was associated with all outcomes, except concerns about using bad coping habits. Grandmothers with less perceived access to care reported greater concern about bad coping habits and remote learning stress, while minority grandmothers reported more financial stress and COVID-19 fear and uncertainty. Findings suggest that the COVID-19 pandemic has compounded the stress experienced by already burdened custodial grandmothers.

